# Recurrent Breast Abscesses due to *Corynebacterium kroppenstedtii*, a Human Pathogen Uncommon in Caucasian Women

**DOI:** 10.1155/2012/120968

**Published:** 2012-09-13

**Authors:** Anne Le Flèche-Matéos, Nicolas Berthet, Fabienne Lomprez, Yolande Arnoux, Anne-Sophie Le Guern, India Leclercq, Ana Maria Burguière, Jean-Claude Manuguerra

**Affiliations:** ^1^Institut Pasteur, Cellule d'Intervention Biologique d'Urgence (CIBU), 75724 Paris, France; ^2^Institut Pasteur, Epidémiologie et Physiopathologie des Virus Oncogènes, 75724 Paris, France; ^3^Centre National de Recherche Scientifique, URA 3015, 75724 Paris, France; ^4^Institut Pasteur, Laboratoire du Centre Médical, 75724 Paris, France

## Abstract

*Background*. *Corynebacterium kroppenstedtii* (*Ck*) was first described in 1998 from human sputum. Contrary to what is observed in ethnic groups such as Maori, *Ck* is rarely isolated from breast abscesses and granulomatous mastitis in Caucasian women. *Case Presentation*. We herein report a case of recurrent breast abscesses in a 46-year-old Caucasian woman. *Conclusion*. In the case of recurrent breast abscesses, even in Caucasian women, the possible involvement of *Ck* should be investigated. The current lack of such investigations, probably due to the difficulty to detect *Ck*, may cause the underestimation of such an aetiology.

## 1. Background

The majority of corynebacteria are normal skin flora. It usually is difficult to distinguish between infection, colonization, and contamination by isolated bacteria. By contrast, our *Corynebacterium kroppenstedtii* (*Ck*) strain was isolated as monomicrobial culture. *Ck* is a lipophilic *Corynebacterium*. Its first isolation was described in 1998 from human sputum [1]. It has also been found in association with inflammatory breast diseases [2]. In 2003, Taylor et al. observed that the incidence of corynebacteria-associated inflammatory breast diseases was much higher in Maori women than women of European descent in whom this type of infection is rare [2]. This species of *Corynebacterium* does not contain mycolic acids and needs lipids for its growth. This is why, the mammary areas, rich in lipids, are favourable to its development and its proliferation. This physiological feature explains the uncommon tissue tropism of this rare bacterium and its ability to efficiently grow in breasts.

## 2. Case Presentation

A 46-year-old Caucasian woman presented recurrent breast abscesses. The patient's medical history revealed that she was a nonsmoker and did not take any kind of medication and she did not have a recent history of breast-feeding. Before symptoms began, there was no sign of breast lesion. Seven months earlier, the first symptom (Month 0 = M0) was a brutal onset of pain in the left breast, with no fever and no other symptom. One month later (M1), the first echography and mammography showed a left external para-areolar mass measuring 24 mm × 16 mm. This hyperechoic mass was surrounded with an hypoechoic and hypervascular halo. At that time, the patient was treated with oral amoxicillin-clavulanic acid 3 g–0.375 g three times a day for 8 days. The patient showed neither clinical nor radiological improvement. After the first course of treatment, the patient was sampled. Cytological and histological analyses of the fine needle aspiration and the biopsy, respectively, did not reveal any sign of malignancy. Three months later (M4), the first surgery allowed to examine the lesion. The patient then received a second course of treatment with amoxicillin-clavulanic acid 3 g–0.375 g three times a day for 10 days. The sampled purulent liquid was sent to a hospital bacterial laboratory for routine and basic analysis, which showed negative results. Six months later (M6), the pain reappeared and the clinical examination revealed para-areolar swelling. A second echography was performed and showed two masses of 30 and 35 mm in the areolar region of the left breast. These masses were hypoechoic and heterogeneous. The lesion was delimited by a thick membrane containing thick pus. Determining the concentration of the C-reactive protein and the blood cell count revealed normal values. One month after (M7) and just before a second surgery, a needle biopsy was performed on one abscess and sent to our laboratory. We isolated and identified, by 16S rRNA gene sequencing as described below, a *Corynebacterium kroppenstedtii* from the clinical sample, which was monomicrobial. The second surgery removed both abscesses, which were sent to a pathology laboratory to search for bacteria including acid-fast bacilli and parasites: none of them were detected. A drain was put in place and left for 10 days. The patient was treated with oral pristinamycin (500 mg three times a day for 15 days) because of its good tissular availability. At M28, the patient has not had any relapse. Therefore, the pristinamycin treatment was efficient to clear the infection, contrary to both treatments by amoxicillin-clavulanic acid despite the strain being sensitive to amoxicillin *in vitro*.

A Gram stain of the pus showed numerous polymorphonuclear leukocytes, but no bacteria by light microscopy. The pus was inoculated onto trypticase soy agar plates containing 5% horse blood and chocolate agar plates (BioMérieux, Marcy l'Etoile, France) and incubated at 37°C in air supplemented with 5% CO_2_. The first isolate was observed after 4 days of incubation. Exactly ten colonies were isolated. The colonies on blood agar were nonpigmented, small, with a diameter of less than 1 mm, smooth, round, matt, and nonhaemolytic. Cells were Gram-positive, nonmotile, non-spore-forming, and club-shaped and arranged in palisades. The results obtained using the API Coryne system identification strip (bioMérieux, Marcy-l'Etoile, France) did not match with any record in the database of the current version (V3.0) for both our isolate and the type strain of *C. kroppenstedtii *(CIP 105744^T^). Antibiotic susceptibility was determined by the disk diffusion method and *E*-test (bioMérieux, Marcy-l'Etoile, France) on Mueller-Hinton agar supplemented with 5% horse blood. The results are shown in Table 1. At present, there are few recommendations about the *Corynebacterium* genus but none about this specific species *C. kroppenstedtii.* With the Clinical and Laboratory Standards Institute (CLSI) guidelines [3], the antimicrobial susceptibility pattern showed our isolate was susceptible to penicillin G, amoxicillin, cefotaxim, minocycline, kanamycin, gentamicin, erythromycin, pristinamycin, clindamycin, vancomycin, rifampicin, linezolid, moxifloxacin, and resistant to trimethoprim-sulfamethoxazole and fosfomycin.

**Figure 1:**
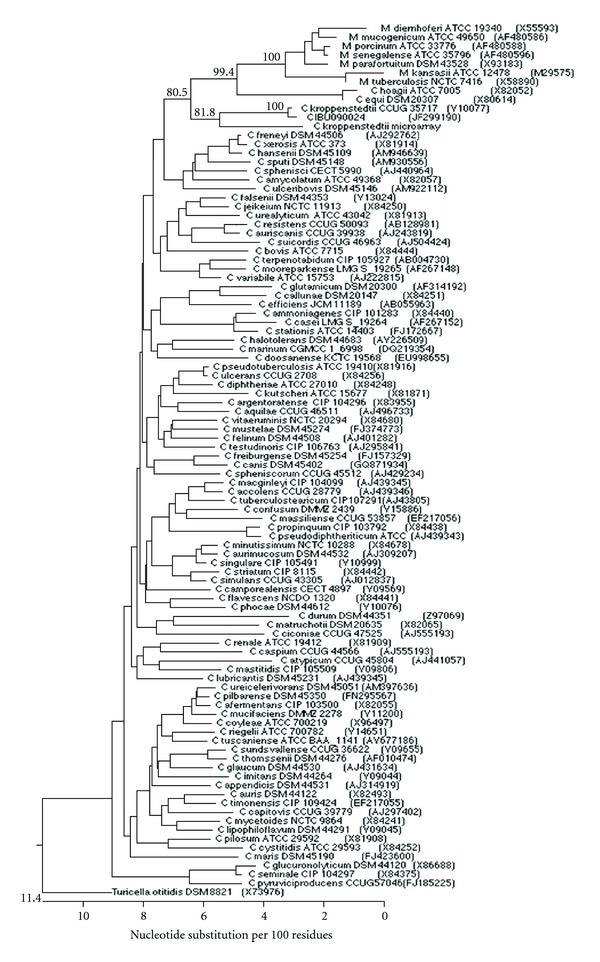
Phylogenetic tree based on 16S rRNA gene sequences of isolate CIBU090024 and representative *Corynebacterium* and *Mycobacterium* species, constructed using the neighbour-joining method. Bootstrap values >50% (based on 1000 replicates) are given at branching points. GenBank accession numbers are given in parentheses. All sequences come from type strains. *Turicella otitidis* DSM 8821^T^ was used as an outgroup.

The complete sequence of the 16S rRNA gene from isolate CIBU090024 was carried out by classical Sanger method as described previously [4]. A total of 1,460 continuous nucleotides were determined. This sequence (GenBank accession number JF299190) was compared to all bacterial sequences available in the GenBank database by using the BLASTN program (http://www.ncbi.nlm.nih.gov/blast/Blast.cgi) and the MegAlign module of the Lasergene software (DNASTAR, Madison, WI, USA). A phylogenetic tree was generated by using the neighbour-joining algorithm [5] (Figure 1). Our isolate was found to have 0.4% divergence with the type strain of *C. kroppenstedtii*. Isolate CIBU090024 thus belongs to the species *Corynebacterium kroppenstedtii.* A resequencing microarray was used in parallel to the bacteriological methods. This molecular tool was developed for the detection of a massive panel of viral and bacterial agents as well as more than 600 genes involved in pathogenicity or antibiotic resistance [6–9]. Bacterial signatures obtained from the isolate confirmed the bacterial identification. No supplementary data was obtained by this way (Data not shown).

## 3. Conclusion

**Figure 2:**
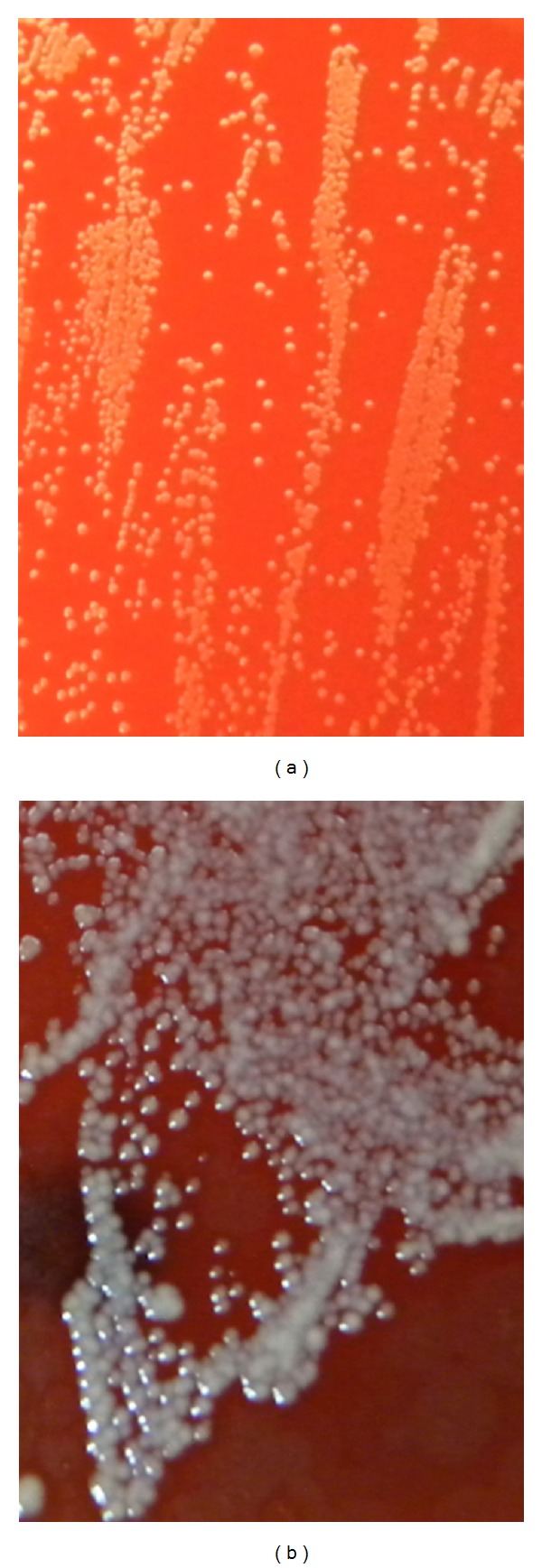
(a) *Corynebacterium kroppenstedtii* (*Ck*) isolation subculture on horse blood agar after 48 h of incubation in aerobic conditions (original magnification, ×25). The first isolation was obtained after 4 days (not shown). (b) *Ck* isolation subculture on horse blood agar supplemented with 1% Tween 80 after 48 h of incubation in aerobic conditions (original magnification, ×25). The colonies were nonpigmented, small (diameter less then 1 mm), smooth, round, matt, and nonhaemolytic.

As a complement to the classical medical bacteriology methods, the contribution of the sequencing of the gene encoding 16S rRNA remains a major tool in the identification of the aetiology of such diseases. *Corynebacterium kroppenstedtii* is a species very difficult to isolate and to identify in the standard conditions of pathology laboratories. The shortest possible lag time between sampling and bacterial culture, the longest possible incubation period necessary before the appearance of the first colonies, and the use of a lipid complement are essential factors for bacterial growth and isolation. This is why 1% Tween 80 was added to horse blood agar (Figure 2) [10]. Isolation is necessary for the successful detection, accurate identification, and antibiotic susceptibility testing of this pathogen. Because it is difficult to isolate and identify, this *Corynebacterium* might be underestimated as the cause of breast abscesses.

## Figures and Tables

**Table 1:** Disk diffusion method compared with *E*-test MICs for type strain of *C. kroppenstedtii* CIP 105744^T^ and our isolate CIBU090024.

Antibiotic	Disk diffusion (mm)		*E*-test MICs (mg/L)	
	*C. kroppenstedtii* CIP 105744^T^	CIBU090024	*C. kroppenstedtii* CIP 105744^T^	CIBU090024
Penicillin G	38	35	0.19	0.47
Amoxicillin	35	45	<0.016	0.094
Cefotaxime	31	40	0.012	0.023
Minocycline	25	33	0.19	0.047
Kanamycin	11	35	3	0.016
Gentamicin	23	40	0.125	0.125
Erythromycin	40	39	0.016	0.25
Pristinamycin	45	40	NA	NA
Clindamycin	26	26	0.094	0.094
Vancomycin	30	32	0.19	0.50
Trimethoprim- sulfamethoxazole	≤6	≤6	0.047	0.064
Fosfomycin	≤6	≤6	1024	>1024
Rifampicin	45	45	0.004	0.002
Linezolid	25	32	<0.016	0.094
Moxifloxacin	25	36	0.032	0.047

NA: nonavailable.
